# Deep learning based automatic segmentation of metastasis hotspots in thorax bone SPECT images

**DOI:** 10.1371/journal.pone.0243253

**Published:** 2020-12-03

**Authors:** Qiang Lin, Mingyang Luo, Ruiting Gao, Tongtong Li, Zhengxing Man, Yongchun Cao, Haijun Wang

**Affiliations:** 1 School of Mathematics and Computer Science, Northwest Minzu University, Lanzhou, Gansu, China; 2 Key Laboratory of China’s Ethnic Languages and Information Technology of Ministry of Education, Northwest Minzu University, Lanzhou, Gansu, China; 3 Key Laboratory of Streaming Computing and Applications, Northwest Minzu University, Lanzhou, Gansu, China; 4 Department of Nuclear Medicine, Gansu Provincial Hospital, Lanzhou, Gansu, China; Charles Sturt University, AUSTRALIA

## Abstract

SPECT imaging has been identified as an effective medical modality for diagnosis, treatment, evaluation and prevention of a range of serious diseases and medical conditions. Bone SPECT scan has the potential to provide more accurate assessment of disease stage and severity. Segmenting hotspot in bone SPECT images plays a crucial role to calculate metrics like tumor uptake and metabolic tumor burden. Deep learning techniques especially the convolutional neural networks have been widely exploited for reliable segmentation of hotspots or lesions, organs and tissues in the traditional structural medical images (i.e., CT and MRI) due to their ability of automatically learning the features from images in an optimal way. In order to segment hotspots in bone SPECT images for automatic assessment of metastasis, in this work, we develop several deep learning based segmentation models. Specifically, each original whole-body bone SPECT image is processed to extract the thorax area, followed by image mirror, translation and rotation operations, which augments the original dataset. We then build segmentation models based on two commonly-used famous deep networks including U-Net and Mask R-CNN by fine-tuning their structures. Experimental evaluation conducted on a group of real-world bone SEPCT images reveals that the built segmentation models are workable on identifying and segmenting hotspots of metastasis in bone SEPCT images, achieving a value of 0.9920, 0.7721, 0.6788 and 0.6103 for *PA* (accuracy), *CPA* (precision), *Rec* (recall) and *IoU*, respectively. Finally, we conclude that the deep learning technology have the huge potential to identify and segment hotspots in bone SPECT images.

## Introduction

Nuclear medicine imaging (also called radionuclide scanning) has been identified as an effective medical modality for diagnosis, treatment, evaluation and prevention of a range of various serious diseases and medical conditions since the early 1990s. Differing from the conventional structural imaging, e.g., Computed Tomography (CT), Magnetic Resonance Imaging (MRI), and Ultrasound imaging, which provides only the structural information about an organ or body part, nuclear medicine imaging allows to reveal both the structural and functional variants in organs and tissues of the body. Thus, nuclear medicine is now an integral part of modern medicine and is extremely prevalent in neurology, oncology and cardiology.

Single Photon Emission Computed Tomography (SPECT) is one of the most commonly used techniques of nuclear medicine imaging which, like Positron Emission Tomography (PET), provides an insight into the physiological processes of the areas of concerns by detecting trace concentrations of radioactively-labeled compounds. In SPECT examination, imaging equipment captures, in a non-invasive manner, the emitted gamma rays from radionuclides that were injected into a patient’s body in advance to generate a map of the inside of a body. The commonly used radiotracers for SPECT imaging include [99mTc] HMPAO (exametazime) for blood flow in the brain, [99mTc] Sestamibi for myocardial perfusion, and [99mTc] MDP (methylene diphosphonate) for bone scanning.

Bones are clinically accepted as the most common sites of metastasis in large number of malignant tumors including prostate and breast cancer. These occupying lesions are seen as areas of increased radioactivity called hotspots in bone SPECT scans. Quantitative bone SPECT scanning has the potential to provide more accurate assessment of disease stage and severity. Specifically, segmenting hotspot plays a crucial role to calculate metrics like tumor uptake and metabolic tumor burden. In the medical image analysis field, image segmentation refers to delineate the boundary of lesions, organs and tissues in the medical images for identifying the target lesion and avoiding normal structures during treatment. Medical image segmentation has been extensively studied in the domains of the traditional machine learning [1–3] and the current deep learning [4–15]. Segmentation of SPECT images has also been a hot research topic in medical image analysis, focusing on the automatic delineation of organs like kidney [16], liver [17, 18], cardiac ventricle [19–21], lung [22–24], tumor like lymphoma [25], and part of body like bladder [26].

However, bone SPECT image segmentation is still in its infancy due to the poor spatial resolution, low signal to noise, and low contrast properties that the bone SPECT imaging has. The size of a whole-body bone SPECT image is 256 × 1024. Currently, only few work has been done on segmenting whole- or partial-body bone structure by utilizing the region growing scheme [27], traditional neural network [28], convolutional neural network combined with active contour model [29], and clustering-based technique [30]. However, active contour model may suffer from edge leakage and high sensitivity to contour initialization [30]. Both clustering and level-set based techniques solely rely on the statistics of intensities in the given image, bringing a significant computation burden [30]. The traditional machine learning based segmentation methods often suffers from insufficient capability and unsatisfied performance for clinical tasks [6].

Deep learning as an emerging machine learning branch has been widely applied in the field of machine vision, speech recognition, natural language processing and other related domains in recent years. Marvelous innovations in deep learning have promoted a variety of popular deep architectures, ranging from the convolutional neural networks (CNNs) [31], recurrent neural networks (RNNs) [32] and deep belief networks (DBNs) [33] to generative adversarial networks (GANs) [34]. Specifically, CNNs are trained end-to-end in a supervised fashion, which extract image features at different abstraction levels by using convolution operators. CNNs are now becoming far more ubiquitous in medical image analysis due to their weight sharing that exploits the intuition of similar structures occurring in various locations in an image. The CNNs-based segmentation models have the potential to automatically learn the features from images in an optimal way. This makes deep learning techniques more popular than those who are built on the traditional machine learning techniques where handcrafted features were extracted by human researchers.

Currently, segmenting metastatic lesions in bone SPECT imaging has not been studies yet. The possible reasons are triple-fold:

First, SPECT imaging especially the whole-body SPECT bone scan is often limited by its poor spatial resolution and low signal-to-noise ratio. It is therefore challenging to display the precise location of a hotspot and its adjacent structures although an abnormal area of increased uptake is noted.

Second, more than one lesion of the same or different primary diseases frequently presents in whole-body bone SPECT images, bringing significant difficulty for correct diagnosis and proper estimation of various diseases.

Last, it is often difficult to build big datasets of bone SPECT scans since the rarity of diseases and patient privacy. Furthermore, imbalanced samples are commonly seen in the bone SPECT imaging because the distribution of bone SPECT images heavily depends on the patients in terms of the type of diseases.

In order to provide a reliable assessment of metastasis in thorax bone SPECT scan, in this work, we propose to develop deep learning based segmentation models that are able to automatically delineate boundary of hotspots in bone SPECT images. Specifically, each of the original DICOM files obtained by SPECT imaging will be first processed to extract the thorax area, followed by image mirror, transition and rotation operations, which contribute to augmenting the original dataset. The famous deep networks including U-Net [35] and Mask R-CNN [36] are then exploited to develop automatic segmentation models by taking advantage of the ability on automatically learning a representation of thorax bone SPECT images that deep learning techniques have. Last, a group of real-world samples of bone SPECT imaging was used to evaluate the developed segmentation models. Experimental results demonstrate that our deep segmentation models are workable and feasible on segmenting hotspots in thorax bone SPECT images, achieving a value of 0.9920, 0.7721, 0.6788 and 0.6103 for PA (accuracy), CPA (precision), Rec (recall) and IoU, respectively.

The main contributions of this work can be concluded as follows.

First, we identify the research problem of automatically delineating metastasis hotspots in bone SPECT images. To the best of our knowledge, this is the first work in deep learning based medical image analysis domain.

Second, we transform the problem into semantic segmentation of thorax bone SPECT images and develop CNNs based segmentation models by taking advantage of the ability of automatically learning the feature representations from images in an optimal way that the deep networks have.

Last, we evaluate the developed deep segmentation models by utilizing a group of real-world bone SPECT images. Experimental results indicate that our models are effective and workable on identifying and segmenting metastasis SPECT images.

The rest of this paper is organized as follows. The utilized data of bone SPECT imaging and the developed deep segmentation models will be detailed in Section 2. Experimental evaluation conducted on real-world SPECT imaging data will be presented in Section 3. And in Section 4, we conclude this work and point out the future research directions.

## Materials and methods

### Bone SPECT image

The used bone SPECT images were collected in the process of diagnosing bone metastases using a Siemens SPECT ECAM imaging equipment in Gansu Provincial Hospital from Jan. 2017 to Dec. 2018. In SPECT examination, the distribution of the intravenous administration of a radiotracer (i.e., 925/740 MBq Tc-99m) to the patient was collected by the equipment.

Patients from different departments were involved in the collected bone SPECT images, including respiratory, thoracic surgery, rheumatology, radiology, oncology, orthopedics, and breast. Inpatients account for the majority of the patients without exclusion of a few of the outpatients. A total of 76 patients aged from 43 to 87 years were clinically diagnosed with metastasis.

Generally, two images, i.e., the anterior and posterior, will be recorded in a SPECT examination if there are no damaged or lost samples. Each bone SPECT image was stored in a DICOM file (.dcm), which is in essence a matrix of radiation dosage that is represented by a 16-bit unsigned integer. The radiation in a wide dosage range makes SPECT images significantly different from the natural images in which the pixel values range from 0 to 255. The size of a whole-body bone SPECT image is 256 (width) × 1024 (height), enabling it to show most of the body of a patient.

Formally, we represent a whole-body bone SPECT image as a matrix *BSI*:

$$BSI=\left[\begin{array}{cccc} rd_{11} & rd_{12} & \ldots & rd_{1m}\\ rd_{21} & rd_{22} & \ldots & rd_{2m}\\ \vdots & \vdots & \ddots & \vdots \\ rd_{n1} & rd_{n2} & \cdots & rd_{nm} \end{array}\right]$$ (1)

where *rd*_*ij*_ (1 ≤ *i* ≤ *m*, 1 ≤ *j* ≤ *n*) represents the radiation dosage, and *m* = 256, *n* = 1024 for the whole-body bone SPECT image.

Finally, a total of 112 samples (i.e., the anterior and posterior) of whole-body bone SPECT images from 76 patients was collected in our dataset.

Since this work focuses solely on automatic segmentation of metastasis hotspots in the thorax area, we need to separate thorax area from a 256 × 1024 whole-body bone SPECT image, followed by image mirror, translation and rotation. These preprocessing operations contribute to augmenting the original dataset simultaneously.

#### Thorax cropping

**Fig 1:**
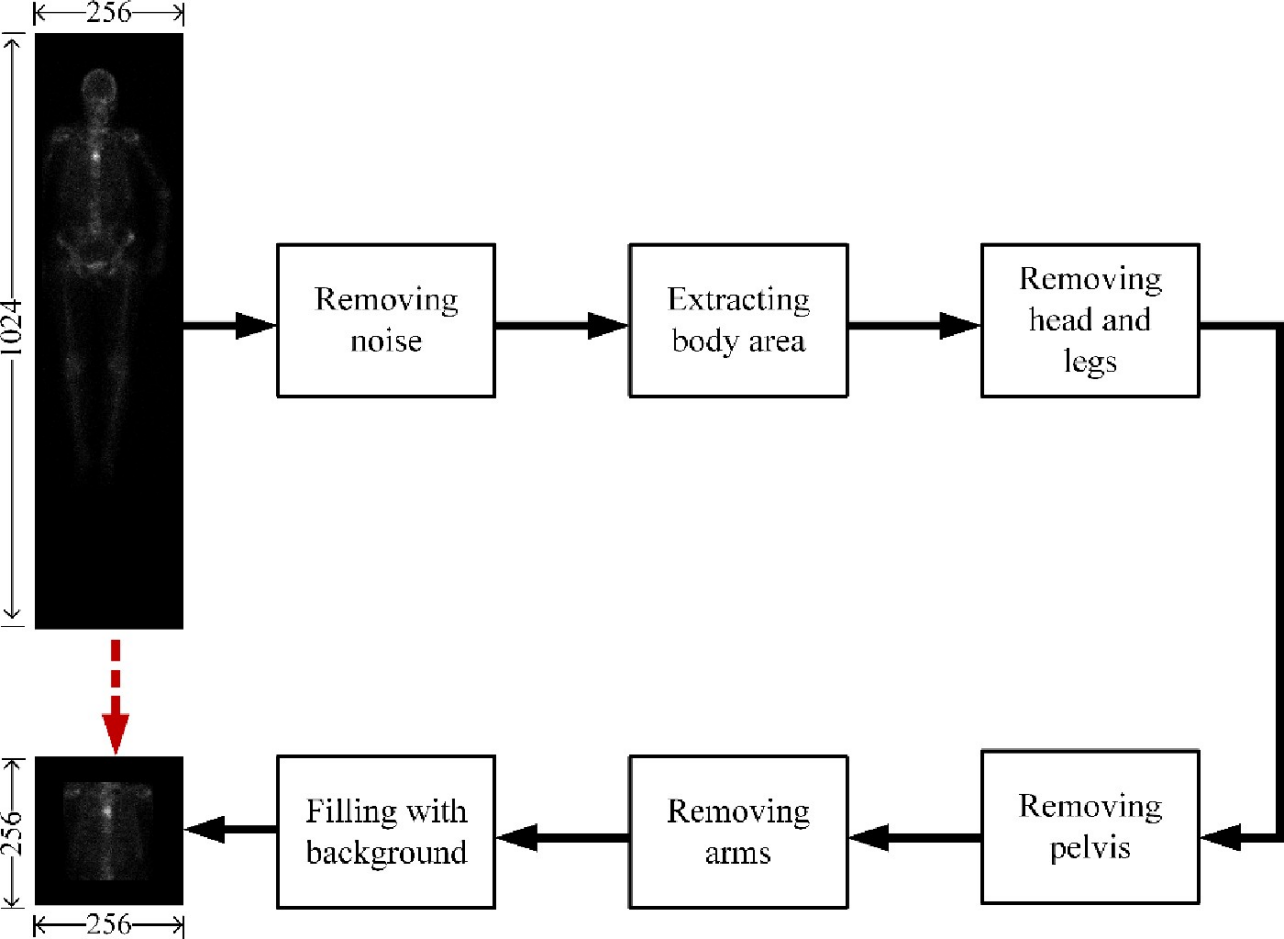
The process of cropping a 256 × 256 thorax bone SPECT image from the original 256 × 1024 whole-body bone SPECT image.

Cropping thorax aims to separate the areas of spine and ribs from the others in a 256 × 1024 whole-body bone SPECT image, to finally extract the thorax area. However, it is often challenging to accurately separate parts of body in a low-resolution bone SPECT image full of noise. Traditional separation methods relying only on information of skeleton structure performs poorly for bone SPECT imaging. The distribution of radiation dosage, on the contrary, should be exploited for adaptively cropping thorax area. Fig 1 depicts the cropping process from an original 256 × 1024 whole-body bone SPECT image to a 256 × 256 thorax bone SPECT image.

*Noise removing*. For a given 256 × 1024 whole-body SPECT image, we globally sweep this image to identify the maximum of radiation dosage outside the body area. The identified maximum is unique for each image and is regarded as the threshold of noise, *thr*_*N*_. Those elements in *BSI* which have radiation dosage of less than *thr*_*N*_ will be set to 0 (i.e., image background). The maximum differs from image to image. This adaptive thresholding based noise removing mechanism is able to remove noise while retaining the information of hotspots.

*Extracting body area*. The SPECT image after removing noise will be further processed to discard those areas above the top of the head and below the toes. On the contrary, other areas (i.e., the left and right of the body) will be left. We call the extracted body area valid area in this paper.

**Fig 2:**
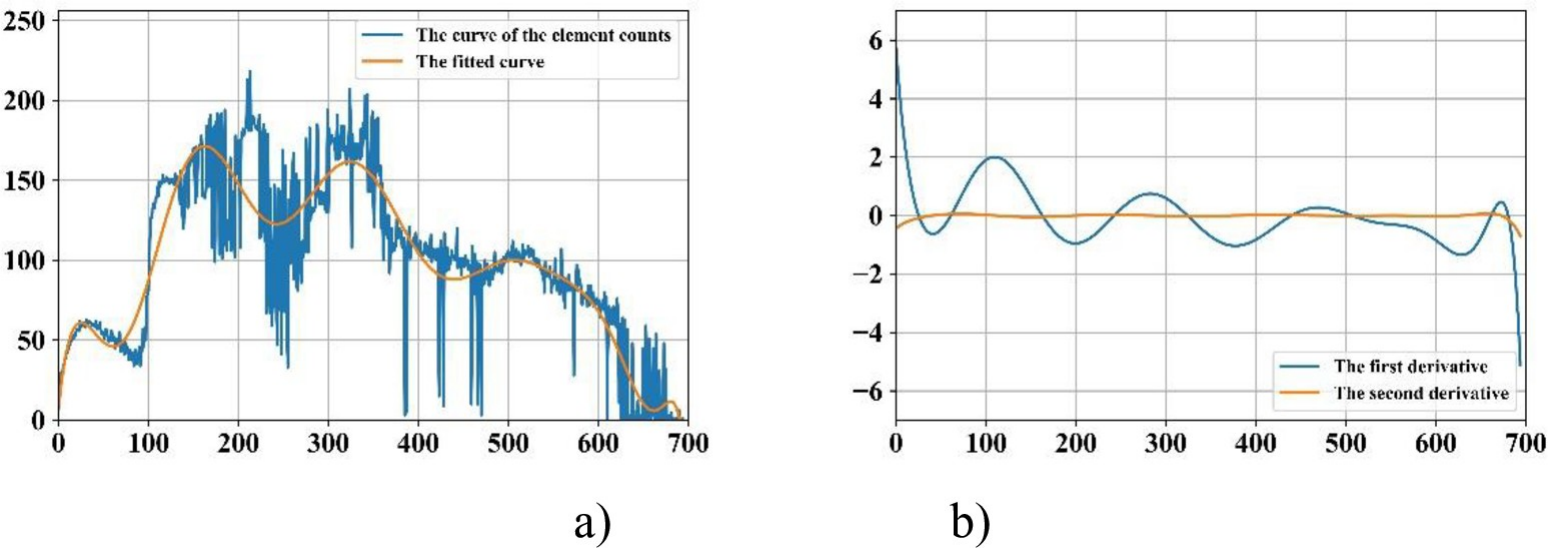
Curve fitting based technique for identifying thorax area. a) The original curve and its fitted one; and b) The curves of the first and second derivatives.

*Removing head and legs*. For the extracted valid area, we count the elements (pixels) from the top down. The generated curve illustrates the presence and intensity of radiation dosage, enabling it to reveal parts of body. For instance, the first three peak points of the fitted curve as illustrated in Fig 2A indicate the beginnings of the head, the right shoulder blades and the right elbow; and the third valley point shows the beginning of the right leg.

*Removing arms and pelvis*. The first and second derivatives plotted in Fig 2B indicate the areas of thorax, arms and pelvis. The area of thorax can then be extracted based on the fact that the width of the trunk is approximately same to the one of area of legs as well as the ratio of height of spine and pelvis is 3: 2.

*Filling with background*. The cropped area of thorax will be enlarged to a size of 256 × 256 by filling the rest areas with background. We call the cropped area of SPECT thorax image or thorax bone SPECT imaging in the sections that follow.

#### Thorax SPECT image augmentation

Deep learning often performs well on the big dataset. We therefore need to augment our dataset by leveraging a series of preprocessing operations on the thorax bone SPECT images based on the following considerations.

- Change in a patient’s position and orientation during the long-time SPECT scan that may take up to 3 hours is inevitable since, for example, the patient is often startled when the bed shifts to the next scanning position. Segmentation models should be robust enough to deal with displacement and tilt in SPECT images.
- The phenomenon of images being not successfully recorded is common in the used dataset. A medical examination has only anterior image, and vice versa, reveals why there are 112 images from 76 patients. Technical approaches need to be applied to handle the missing of bone SPECT images.

Preprocessing techniques including mirror, translation, and rotation are used to cope with the problems above, which extends the dataset simultaneously.

*Image mirror*: Horizontal mirror is used to reverse a SPECT thorax image right-to-left along the vertical center line of the image. Given an input point (*x*_*i*_, *y*_*i*_), its output (*x*_*o*_, *y*_*o*_) after mirroring can be mathematically represented as follows.

$$\left[\begin{array}{c} x_{o}\\ y_{o}\\ 1 \end{array}\right]=\left[\begin{array}{ccc} -1 & 0 & w\\ 0 & 1 & 0\\ 0 & 0 & 1 \end{array}\right]\left[\begin{array}{c} x_{i}\\ y_{i}\\ 1 \end{array}\right]$$ (2)

*Image translation*. A SPECT image is translated by +*t* or -*t* pixels in either horizontal or vertical direction. For each SPECT image, *t* is randomly assigned with an integer within the range [0, *t*_*T*_]. By contrary, *t*_*T*_ is statistically determined according to the distribution of radiation dosage in all images. A value of 10 (4) for *t*_*T*_ is workable in the experiments for horizontal (vertical) translation. Formally, for an input point (*x*_*i*_, *y*_*i*_), its output (*x*_*o*_, *y*_*o*_) of the horizontal or vertical translation can be mathematically represented in Eq 3.

$$\left[\begin{array}{l} x_{o}\\ y_{o} \end{array}\right]=\left[\begin{array}{cc} 1 & 0\\ 0 & 1 \end{array}\right]\left[\begin{array}{c} x_{i}\\ y_{i} \end{array}\right]+\left[\begin{array}{c} \Delta x\\ \Delta y \end{array}\right]$$ (3)

**Fig 3:**
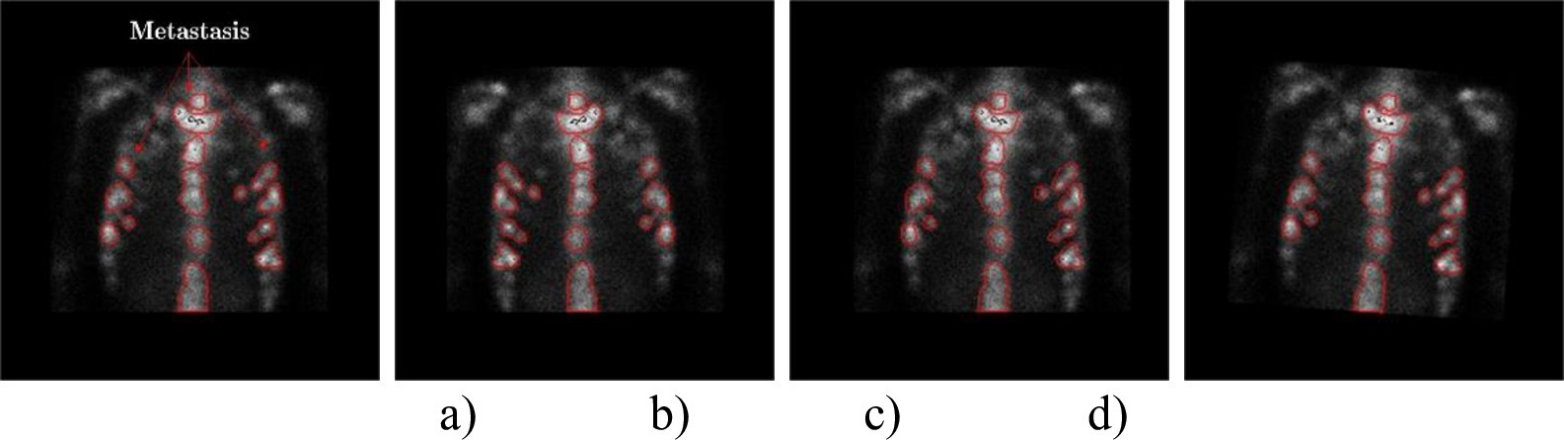
An example of preprocessing thorax bone SPECT image. a) The original thorax bone SPECT image; b) The horizontally mirrored image; c) The horizontally translated image by + 6 pixels; and d) The rotated image by +5°.

We can see from Fig 3 that the information of hotspots in the translated images keep perfectly.

*Image rotation*. A SPECT image is rotated by *r* degrees in either left or right direction around its geometric center. For each SPECT image, *r* is randomly assigned with an integer within the range [0, *r*_*T*_]. Similarly, *t*_*T*_ is statistically determined according to the distribution of radiation dosage in all images. Similarly, the output (*x*_*o*_, *y*_*o*_) after rotating an input point (*x*_*i*_, *y*_*i*_) can be mathematically represented as follows.

$$\left[\begin{array}{c} x_{o}\\ y_{o} \end{array}\right]=\left[\begin{array}{cc} \cos\theta & \sin\theta \\ -\sin\theta & \cos\theta \end{array}\right]\left[\begin{array}{c} x_{i}\\ y_{i} \end{array}\right]$$ (4)

A value of 5° for *r*_*T*_ is workable in the experiments for both left and right rotation. An example of rotating a given image to the right direction by 5^o^ is depicted in Fig 3D.

The generated images obtained by applying the pre-processing operations above will be added to the original dataset of bone SPECT images. Finally, a total of 2 280 samples are contained in the augmented dataset. Table 1 outlines the used data in this work.

**Table 1:** An overview of the used data of SPECT images.

Dataset	Sample	Training sample	Testing sample
**The original**	112	–	–
**The augmented**	2 280	1 830	450

The subsequent section describes the process of labelling bone SPECT images for obtaining ground truth in the experiments for each image.

#### SPECT image labelling

**Fig 4:**
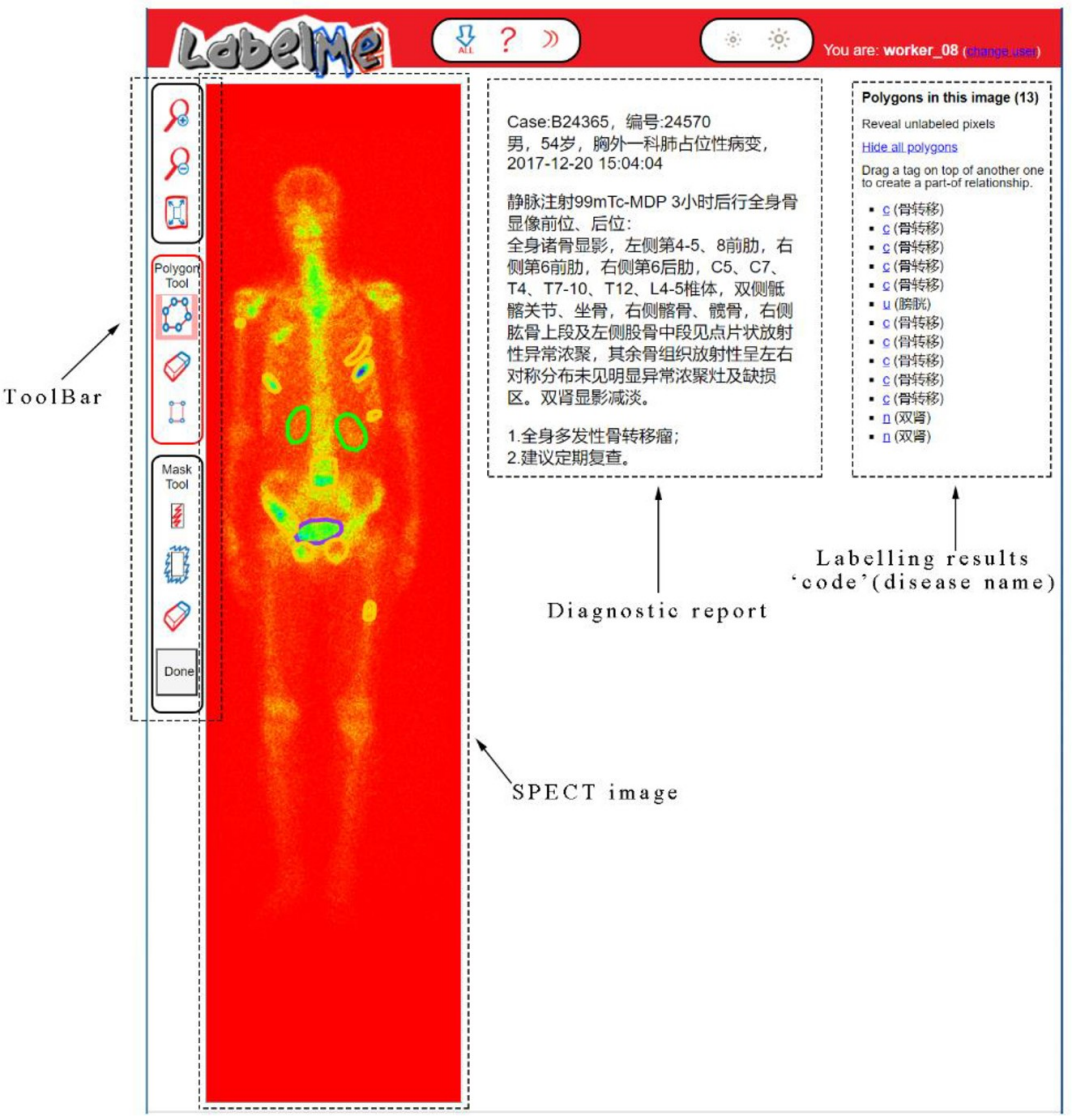
Labelling SPECT image using the LableMe based annotation system.

In supervised learning domain, image labelling plays crucial role for training a reliable deep learning based segmentation model. However, it is more time-consuming and laborious to label a SPECT image due to its low spatial resolution. Based on the openly available tool LableMe released by MIT (http://labelme.csail.mit.edu/Release3.0/), we develop a SPECT image annotation system in this work (see Fig 4).

As depicted in Fig 4, the DICOM file of a whole-body bone SPECT image and the diagnostic report in text format were imported into LableMe in advance. Three nuclear medicine doctors in our research group are then asked to manually label areas on the visual presentation of DICOM file (RGB format is currently used but not limited to this) with a shape tool (e.g., polygon and rectangle) in the toolbar. The labelled area will be annotated with a self-defined code combined with the name of disease or body part. The results of manual annotation for all SPECT images serve as ground truth in the experiments and form an annotation file together, which will be fed into the segmentation models.

Specifically, the bone SPECT image annotation process is performed by three nuclear medicine doctors independently according to the diagnosis report. If the majority of the doctors (i.e., at least two of them) think that an image is abnormal (i.e., at least one lesion of a disease presents in it), it is labeled as a malignant one; otherwise, it is labeled as a benign image. For our augmented dataset consisting of 2 280 thorax bone SPECT images diagnosed with metastasis, three doctors manually delineate hotspots using the polygon tool of the LabelMe annotation system. It is worth noticing that in our dataset, an image may contain multiple lesions but they belong to the same instead of different diseases.

The used bone SPECT images were de-identified before the authors received the data. The fully anonymised image data was received by the authors on 28 August, 2020. A requirement for informed consent was waived for this study because of the anonymous nature of the data. The study was approved by the Ethics Committee of Gansu Provincial Hospital (Lot No.: 2020–199).

### Segmentation models

In this work, we develop several deep segmentation models based on the mainstream CNN networks, which will be detailed below. Furthermore, in order to provide a comparison of performance on segmenting hotspots in bone SPECT images between the current deep learning and traditional machine learning techniques, we also construct a clustering based segmentation model.

#### U-Net based segmentation

**Fig 5:**
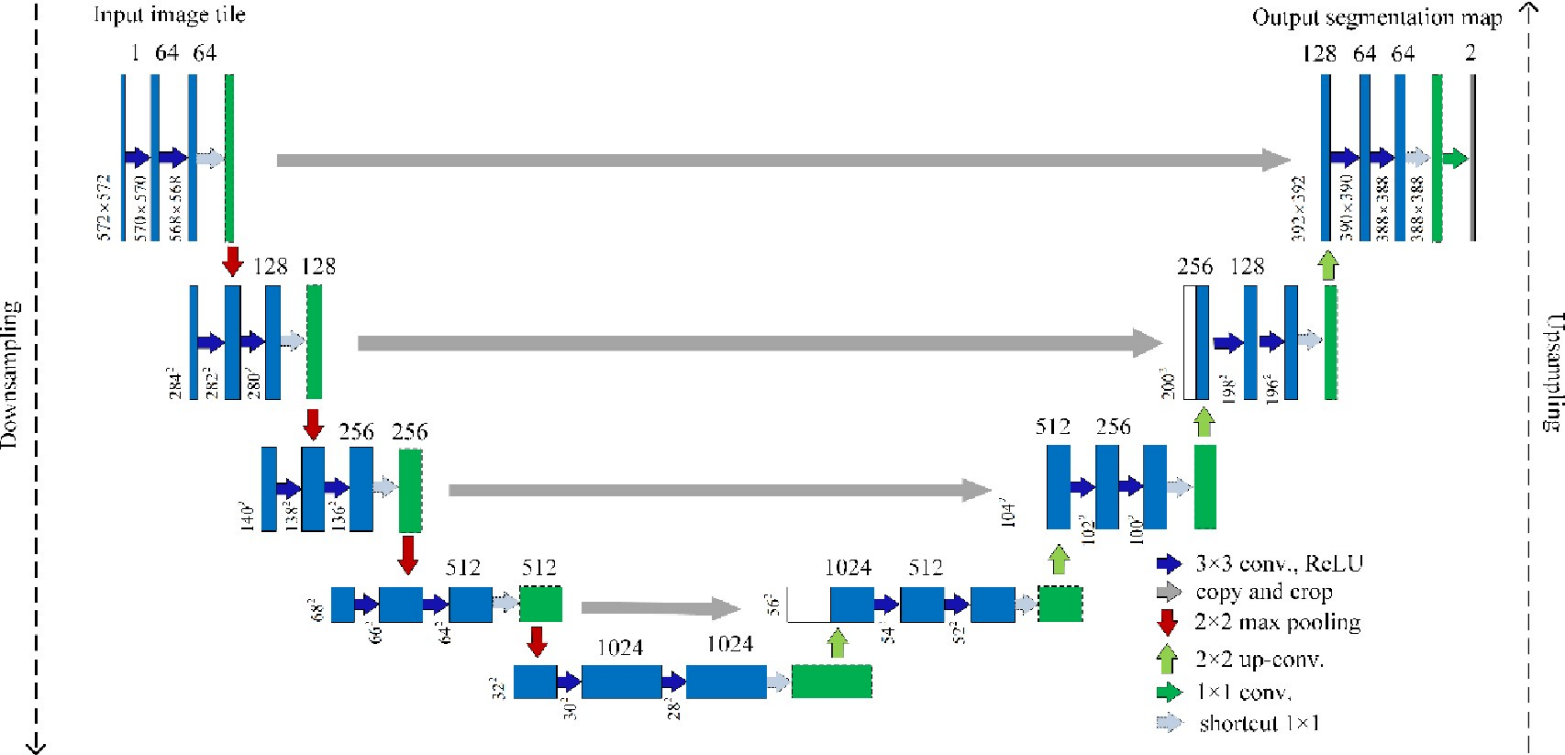
The architecture of the U-Net based segmentation network with shortcut module.

The U-Net [35] as a deep segmentation model was proposed solely for biomedical image segmentation on very few images. As illustrated in Fig 5, the architecture of U-Net consists of a contraction path (i.e., downsampling) for capturing context and a symmetric expansion path (i.e., upsampling) for precise localization.

- Contraction path: This path accounts for downsampling an imputed image, which consists of the repeated application of two 3 × 3 convolutions, followed by a ReLU and a 2 × 2 max pooling with stride 2. The number of feature channels at each downsampling step will be doubled.
- Expansion path: This path consists of an upsampling of the feature map, followed by a 2 × 2 convolution that halves the number of feature channels, a concatenation with the correspondingly cropped feature map from the contracting path, and two 3 × 3 convolutions, each followed by a ReLU. At the final layer, a 1 × 1 convolution is used to map each 64-component feature vector to the desired number of classes.

**Fig 6:**
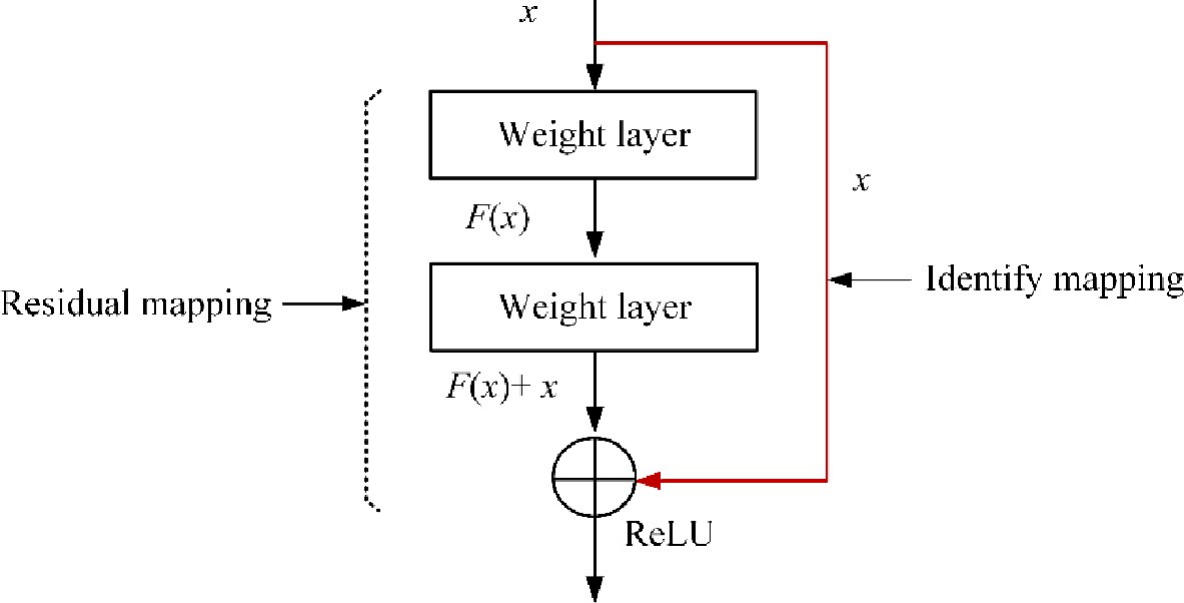
The structure of a residual module.

Based on U-Net network, we develop two segmentation models in this work. Specifically, the first one is built by directly using the standard U-Net. The second one is developed by adding a 1 × 1 residual module after the repeated two convolutions in U-Net (see shortcut in Fig 5), aiming to reduce the training parameters and training time. Fig 6 depicts the structure of residual module. For the feature **F**(*x*) of an input *x*, it residual mapping *y* can be mathematically represented as follows:

y=f(F(x,w)+H(x)) (5)

where *w* is a parameter and *f* is the ReLU function.

Specifically, **F**(*x*) = *x* denotes the identify mapping in the residual module.

The U-Net network with residual module has the potential to deal with the degradation and gradient vanishing problem. In this paper, the improved U-Net after adding residual module is named *U-Net-Res*.

#### Mask R-CNN based segmentation

**Fig 7:**
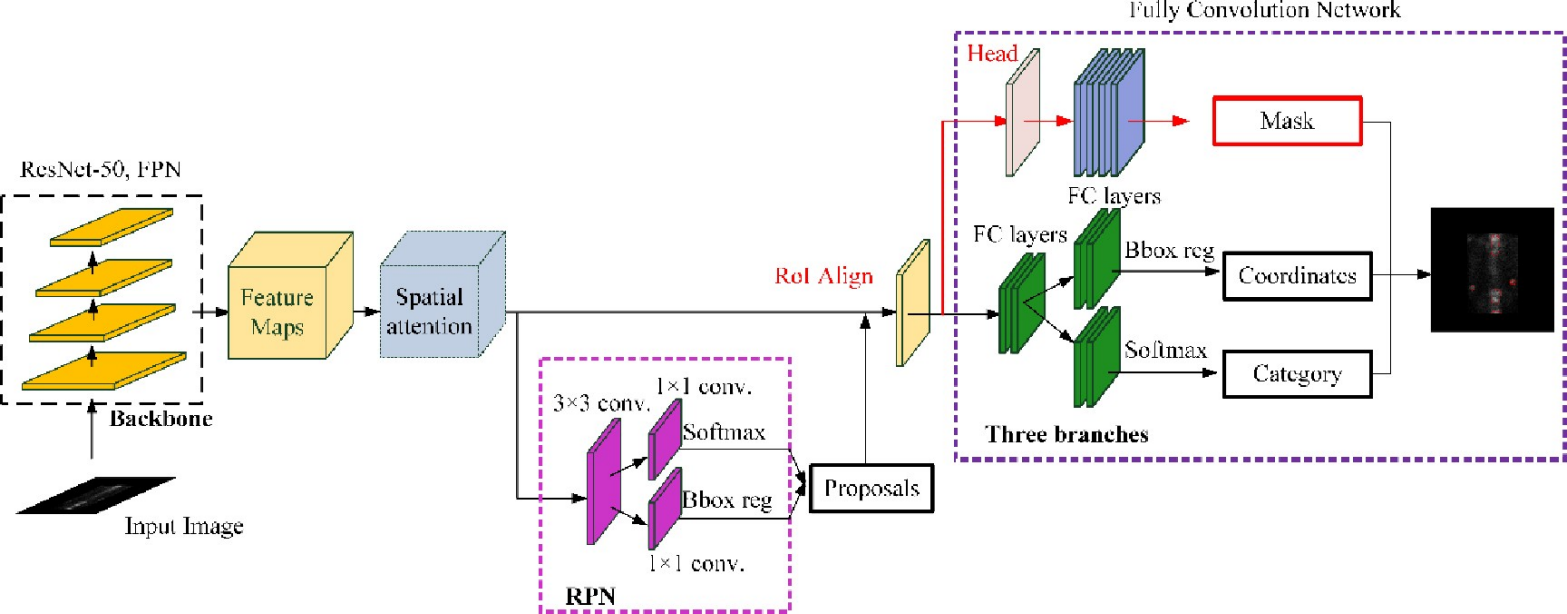
The architecture of the Mask-RCNN based segmentation network with spatial attention module.

Mask R-CNN [36] is an object instance segmentation network, which is proposed by adding a branch for predicting an object mask into the Faster R-CNN [37]. Mask R-CNN is able to detect objects in an image while simultaneously generating a high-quality segmentation mask for each instance. The network architecture of Mask R-CNN is depicted in Fig 7, where the backbone is used for feature extraction and the network head comprises of object detection and segmentation parts.

**Fig 8:**
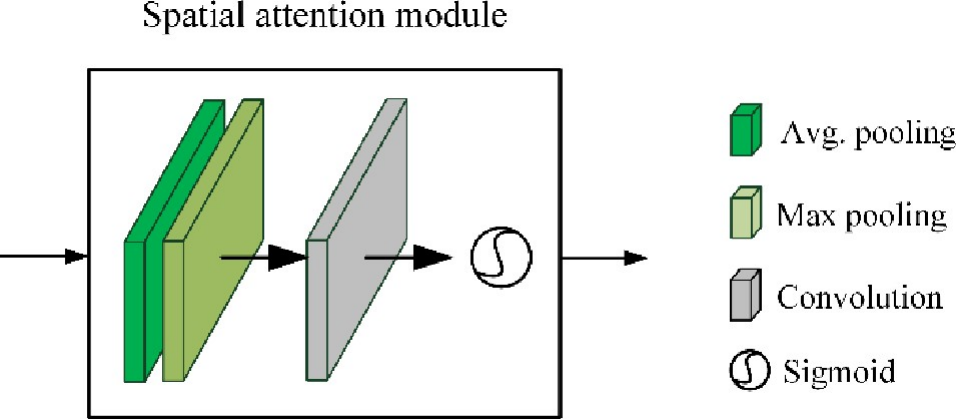
The structure of spatial attention module.

Based on Mask R-CNN, we develop two segmentation models in this work. Specifically, the first one is built by directly using the standard Mask R-CNN network, where a 50-layer ResNet network is used in the backbone. The second one is developed by adding the spatial attention mechanism into the standard Mask R-CNN network. The structure of the added spatial attention mechanism is depicted in Fig 8, consisting of a 1 × 1 average pooling layer, a 1 × 1 max pooling layer, a 7 × 7 convolutional layer, and a Sigmoid non-linearity operation.

The spatial attention module is used to help Mask R-CNN focus on those more important areas on the feature maps by considering the spatial importance information. For the inputted feature map **F**, the output of spatial attention module is a 1 × *H* × *W* feature matrix **M**_*s*_(**F**):

$$M_{s}(F)=\sigma \left(f^{7\times 7}\left(\left[AvgPool(F);MaxPool(F\right)\right)\right)$$ (6)

where *σ* is the Sigmoid function, *f* ^7×7^ is the 7 × 7 convolutional operation; and *AverPool* and *MaxPool* denotes the average pooling and max pooling, respectively.

The improved Mask R-CNN after adding spatial attention mechanism is named *Mask R-CNN-Att* in this paper. The input to the networks above is a fixed-size 256 × 256 thorax bone SPECT image and the output is a (group of) hotspot(s) described by the category, coordinates, and mask.

#### Clustering based segmentation

Clustering has been seen as the first technique for segmentation of natural images since 1990s due to its simplicity and efficiency [38]. *K*-means clustering is one of the most commonly utilized centroid-based clustering techniques, where *K* that is manually specified refers to the number of clusters.

After randomly initializing the center of the *K* clusters, the *K*-means clustering algorithm works by iteratively executing the following two steps until the stopping criteria is met [39]:

**Step 1:** Attribute the closest cluster to each data point

**Step 2:** Set the position of each cluster to the mean of all data points belonging to that cluster

Generally, there are three stopping criteria that can be adopted to stop the *K*-means algorithm:

1. The points in the same cluster remain without adding and removing.
2. The centroids of the newly formed clusters do not change, which can be approximately measured by the optimization objective function defined in Eq. 7.
  $$\underset{S}{\arg\;\min}\sum_{i=1}^{k}{\sum_{x\in S_{i}}\left\|x-\mu_{i}\right\|}^{2}$$ (7)
  where *μ*_*i*_ is the mean of points in the *i*-th cluster *S*_*i*_.
3. The maximum number of iterations are reached.

**Fig 9:**
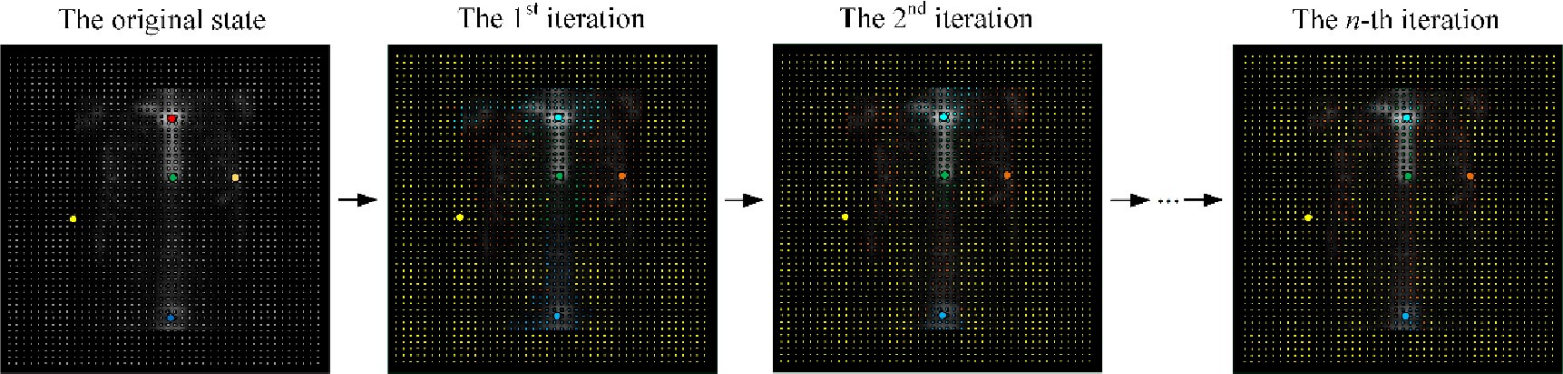
An example of *K*-means clustering based hotspot segmentation with thorax bone SPECT image with *K* = 5.

How to specify a proper *K* becomes the key for *K*-Means clustering algorithm. In this work, we choose the different *K* and pick the one that makes the most sense for our hotspot segmentation task. Fig 9 provides an example of *K*-Means clustering based hotspot segmentation of thorax bone SPECT image, where the centroid (marked by the solid point) of each cluster changes as the iterative execution of the algorithm.

## Results

In this section, we provide an empirical evaluation of the developed deep learning based segmentation models using a real-world dataset consisting of 2 280 thorax bone SPECT images, i.e., 1 830 samples for training and 450 samples for testing the built segmentation models.

### Experimental setup

The evaluation metrics we utilize are *PA* (accuracy), *CPA* (class pixel accuracy), *Rec* (recall), and IoU (intersection over onion). In practice, a classified pixel falls into one of the four categories:

- True Positive (*TP*), which correctly identifies a malignant pixel as positive;
- False Positive (*FP*), which incorrectly identifies a benign pixel as positive;
- False Negative (*FN*), which incorrectly identifies a malignant pixel as negative; and
- True Negative (*TN*), which correctly identifies a benign pixel as negative.

Accordingly, we define *PA*, *CPA*, *Rec*, and IoU in Eqs 8–11.

$$PA=Accuracy=\frac{TP+TN}{TP+TN+FP+FN}$$ (8)

$$CPA=Precision=\frac{TP}{TP+FP}$$ (9)

$$Rec=\frac{TP}{TP+FN}$$ (10)

$$IoU=\frac{TP}{TP+FP+FN}$$ (11)

The parameter setting is: Optimizer = SGD (stochastic gradient descent), Learning rate = 0.001, Batch size = 16, and Epoch = 100. We use 70% of the samples (i.e., 1 830 samples) for training and the rest (i.e., 450 samples) for testing the developed segmentation models.

The experiments are run in Tensorflow 2.0 on an Inter Xeon(R) Silver 4110 PC with 16 Kernels 62GB RAM running Ubuntu 16.04 equipped with GeForce RTX2080 × 2.

### Experimental results

**Fig 10:**
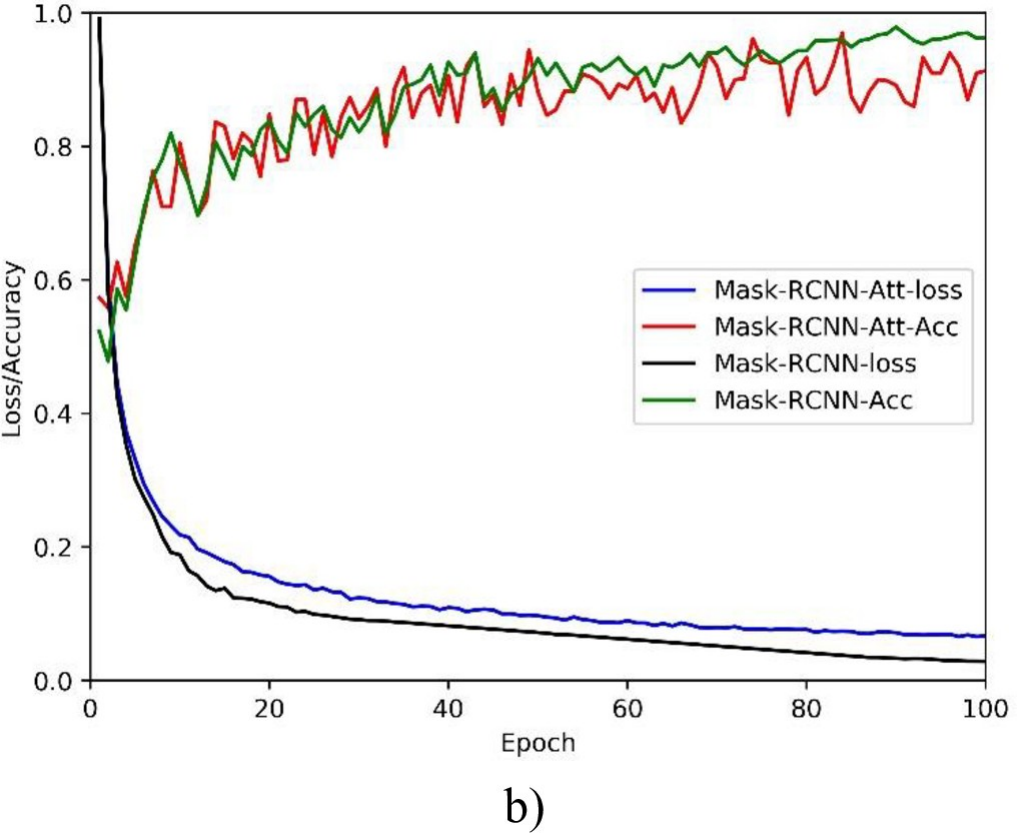
PA and loss curves of two segmentation models. a) U-Net; and b) Mask R-CNN.

Fig 10 demonstrates the training processes of the developed deep segmentation models on segmenting hotspots in thorax bone SPECT images, in terms of the PA (accuracy) and loss metrics. We can see from the accuracy and loss curves in Fig 10 that, the improved U-Net model U-Net-Att achieves the best segmentation performance. This can be further proved by the quantitative results of various evaluation metrics as shown in Table 2.

**Table 2:** Experimental results on evaluation metrics for 2 280 samples of thorax bone SPECT imaging.

Segmentation model	*PA*	*CPA*	*Rec*	IoU
**U-Net**	**0.9920**	0.7624	0.6726	0.5941
**U-Net-Res**	0.9818	**0.7721**	**0.6788**	**0.6103**
**Mask R-CNN**	0.9724	0.7292	0.6508	0.5544
**Mask R-CNN-Att**	0.9676	0.6958	0.6348	0.5427

From the high *PA* we can see that our deep segmentation models are workable to identify both the metastasis and background pixels in thorax bone SPECT images. However, relatively low *CPA* and *Rec* have been obtained by the models, which in turn lead to the low *IoU*. The possible reasons can be concluded as follows.

- The proportion of metastasis areas on the whole image is very low since there is often smaller number of hotspots in most of the thorax bone SPECT images. Thus, the correctly classified background pixels mainly contribute to the high *PA*.
- The low resolution of SPECT imaging brings a huge challenge for oncologists to precisely delineate hotspots in bone SPECT images. The annotation errors mainly contribute to the misclassification of metastasis pixels. As a result, all models achieve the high *PA* but the relatively low *CPA* and *Rec*.

On the whole, the improved model U-Net-Res outperforms the others. We can conclude that the U-Net has the potential for hotspot segmentation task of bone SPECT images and the residual operation is helpful for U-Net focusing on the important areas that may denote the hotspots in bone SPECT images.

**Fig 11:**
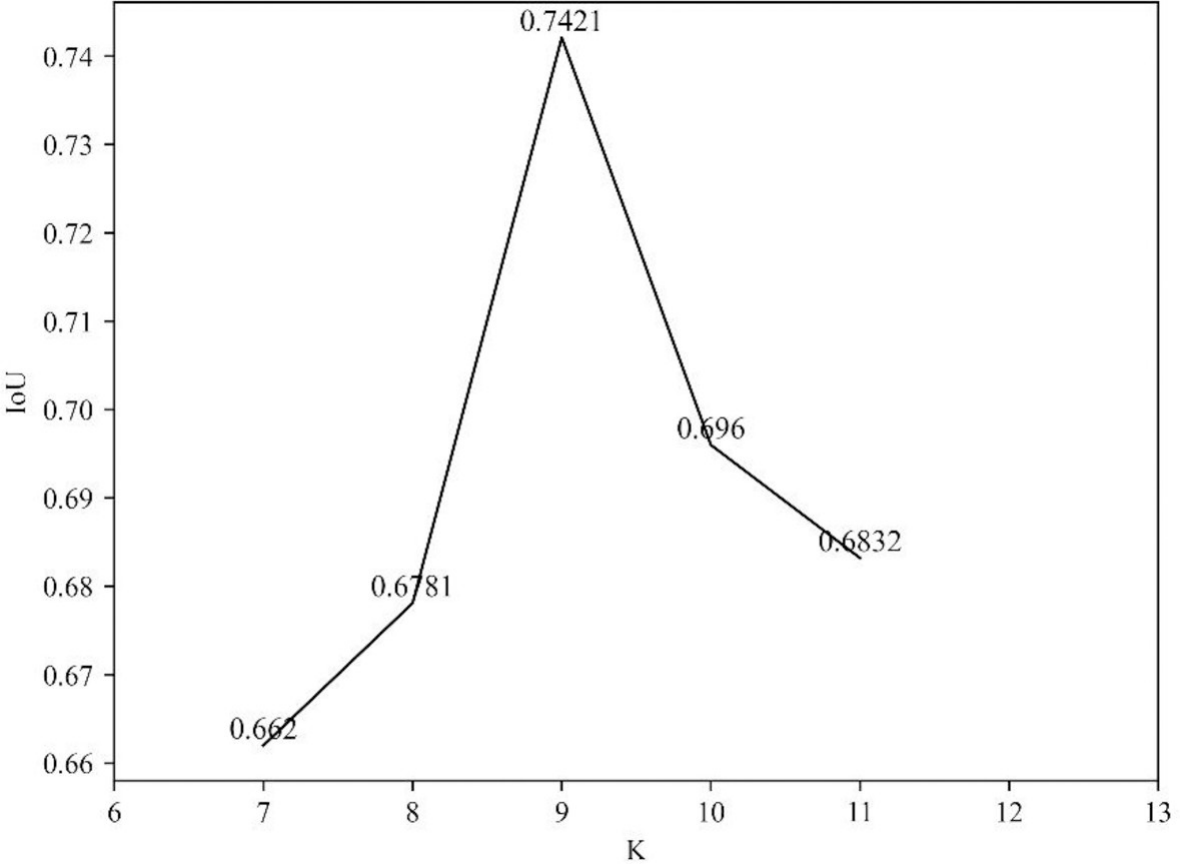
The IoU values obtained by *K*-means based segmentation model for different *K*.

In order to provide a comparison between the developed deep models and the clustering based method, Fig 11 shows the IoU obtained by *K*-means clustering based segmentation where *K* is assigned with different values. We can see that higher values of IoU are achieved by the *K*-means clustering algorithm than the deep models. In the best case of *K* = 9, the *K*-means based segmentation obtains a value of 0.7421 for IoU.

However, the traditional clustering algorithm segments a hotspot by leveraging only the visual features (i.e., brightness and color) of SPECT images. This may lead to two problems as follows.

**Fig 12:**
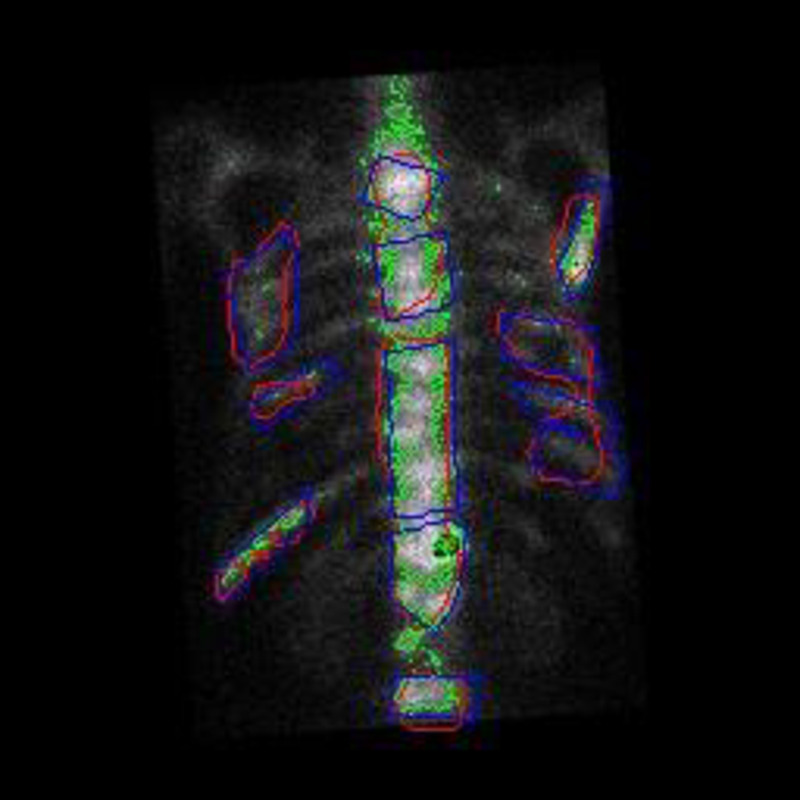
A comparison of *K*-means based (green), U-Net based (blue) and the manually labelled (purple) segmentation results.

- First, any local areas that have different visual features can be separated well from the background. But the categories that these areas belong to are still unknown. Therefore, the traditional clustering techniques are not suitable for multi-disease segmentation task. In contrast, with the extracted semantic features from medical images, the deep learning techniques are able to not only delineate the boundary of lesions but also to identify the categories of these lesions.
- Second, the clustering based segmentation often has high false alarm rate. For example, most isolate points and some dense areas are incorrectly detected as lesion areas in Fig 12 because of their high brightness. Actually, the deep models take the symmetric relation of bone structure into the semantic segmentation of bone SPECT image.

Therefore, deep learning based approaches are more suitable for identifying and delineating metastatic hotspots than the traditional machine learning algorithms due to their ability on automatically learning feature representations from SPECT images in an optimal way.

**Fig 13:**
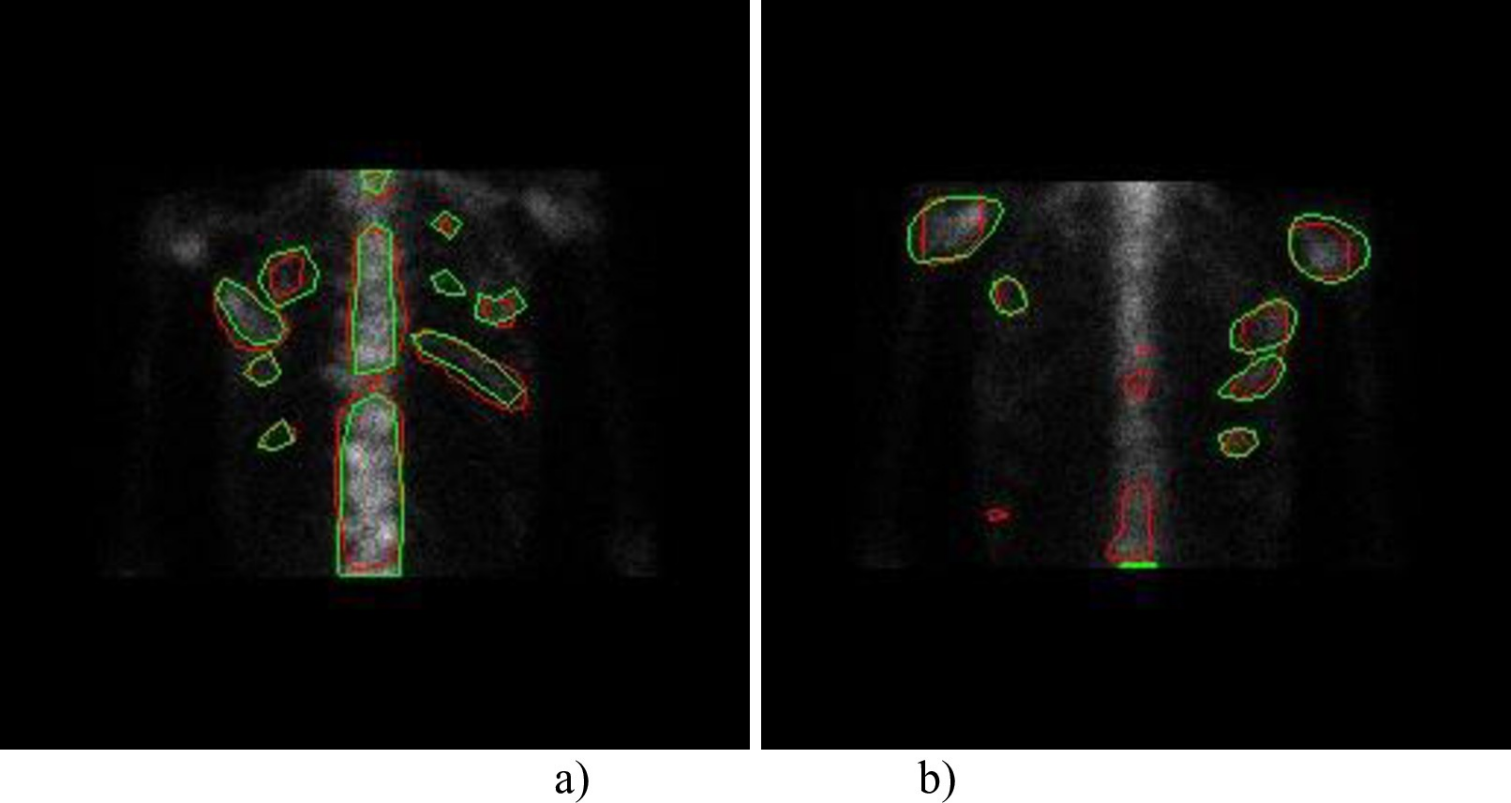
A visualization of hotspots segmented by U-Net-Res model for two thorax bone SPECT images (the model segmented results marked with green and the manually labeled ones marked with red). a) The best case; and b) The worst case.

From the visualization as depicted in Fig 13 we can see that: 1) for the best case, the U-Net-Res model almost identifies all hotspots as well as the differences between the model segmented areas and the manually delineated ones are mainly derived from the errors of the oncologists’ manual delimitation; and 2) for the worst case, apart from the differences mentioned above, some manually labeled areas are not successfully identified by the deep segmentation model. The possible reason is the insufficient samples in the sub-category of bone SPECT images that these samples belong to.

In a nutshell, the developed deep segmentation models, especially the U-Net-Res model, are workable for segmentation of hotspots in bone SPECT images. The deep learning technique has the potential to be used as a type of emerging techniques for automated hotspot or lesion segmentation task.

## Conclusions and future work

Focusing on the automatic semantic segmentation of hotspots in bone SPECT images, in this work, we have developed several deep learning based segmentation models. First, the original whole-body bone SPECT images were processed to extract thorax areas and augment the size of dataset by utilizing mirror, translation and rotation operations. Second, the famous deep networks including U-Net and Mask R-CNN were chosen as the basis to develop our segmentation models by adding new function modules. Last, a group of real-world samples of bone SPECT images were used to evaluate the built models. The experimental results have demonstrated that our models are workable for identify both the hotspot and background pixels.

In the future, we plan to extend our work in the following directions.

First, we intend to collect more real-world SPECT bone scan images to comprehensively evaluate the developed deep segmentation models. Accordingly, optimization and improvement will be done for developing more robust, effective, and efficient computer-aided diagnosis (CAD) system.

Second, we attempt to develop multi-class, multi-disease models to segment hotspots of a great number of diseases in SPECT thorax images.

Last, we plan to build self-defined deep networks, targeting exclusively at segmentation of bone SPECT images for enlarging the current research domain of medical image analysis.

## Supporting information

S1 Fig The process of cropping a 256 × 256 thorax bone SPECT image from the original 256 × 1024 whole-body bone SPECT image. (TIF)

S2 Fig Curve fitting based technique for identifying thorax area. a) The original curve and its fitted one; and b) The curves of the first and second derivatives.
(TIF)

S3 Fig An example of preprocessing thorax bone SPECT image. a) The original thorax bone SPECT image; b) The horizontally mirrored image; c) The horizontally translated image by + 6 pixels; and d) The rotated image by +5°.
(TIF)

S4 Fig Labelling SPECT image using the LableMe based annotation system. (TIF)

S5 Fig The architecture of the U-Net based segmentation network with shortcut module. (TIF)

S6 Fig The structure of a residual module. (TIF)

S7 Fig The architecture of the Mask-RCNN based segmentation network with spatial attention module. (TIF)

S8 Fig The structure of spatial attention module. (TIF)

S9 Fig An example of K-means clustering based hotspot segmentation with thorax bone SPECT image with K = 5. (TIF)

S10 Fig PA and loss curves of two segmentation models. a) U-Net; and b) Mask R-CNN.
(TIF)

S11 Fig The IoU values obtained by K-means based segmentation model for different. (TIF)

S12 Fig A comparison of K-means based (green), U-Net based (blue) and the manually labelled (purple) segmentation results. (TIF)

S13 Fig A visualization of hotspots segmented by U-Net-Res model for two thorax bone SPECT images (the model segmented results marked with green and the manually labeled ones marked with red). a) The best case; and b) The worst case.
(TIF)

S1 Table An overview of the used data of SPECT images. (DOCX)

S2 Table Experimental results on evaluation metrics for 2 280 samples of thorax bone SPECT imaging. (DOCX)
