# Supplementary figures and images for: Deep learning based automatic segmentation of metastasis hotspots in thorax bone SPECT images
Source: PLoS One. 2020 Dec 3;15(12):e0243253. doi: 10.1371/journal.pone.0243253 (PMC7714246; doi:10.1371/journal.pone.0243253)

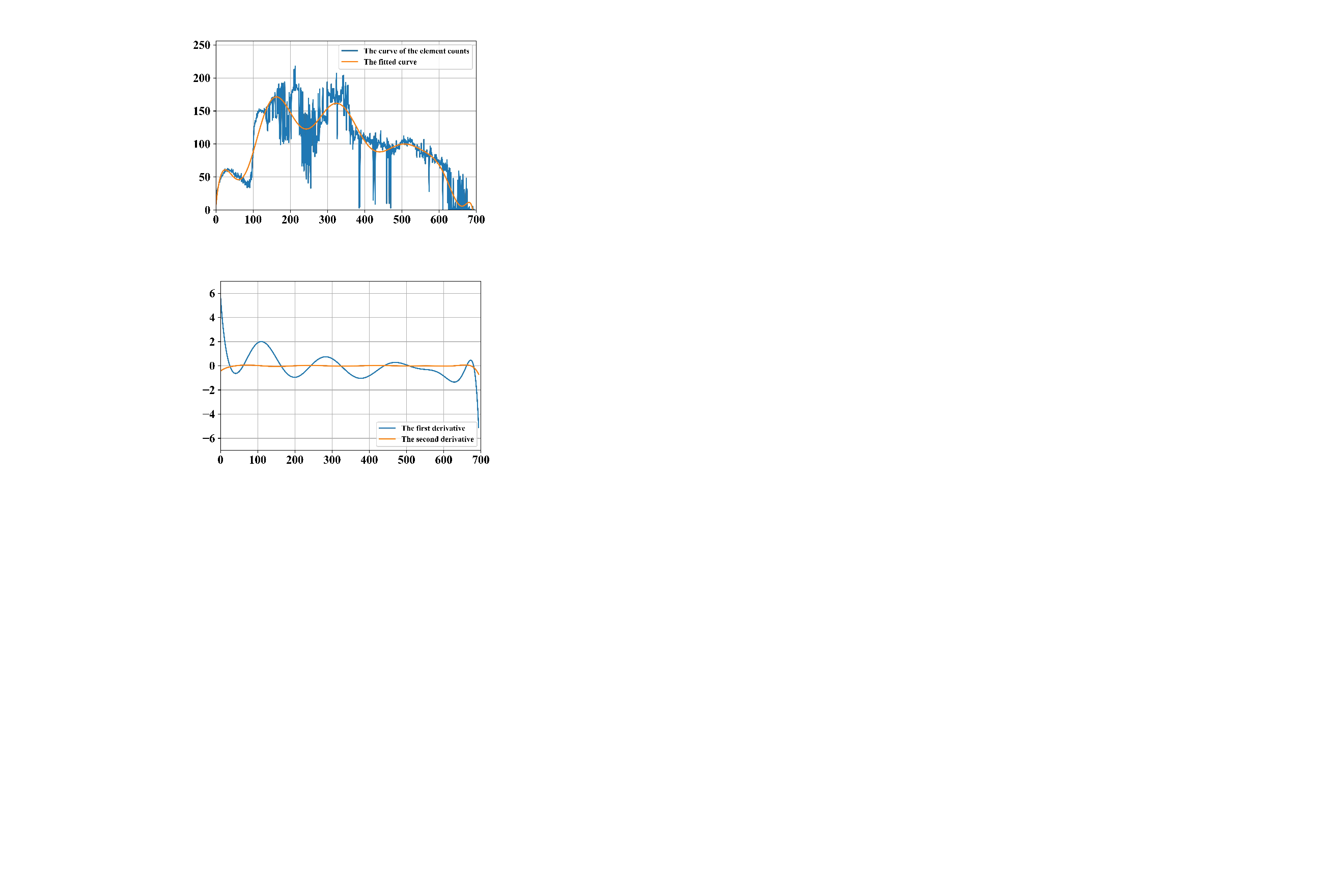

Supplement: S2 Fig — a) The original curve and its fitted one; and b) The curves of the first and second derivatives. (TIF) [file pone.0243253.s002.tif]

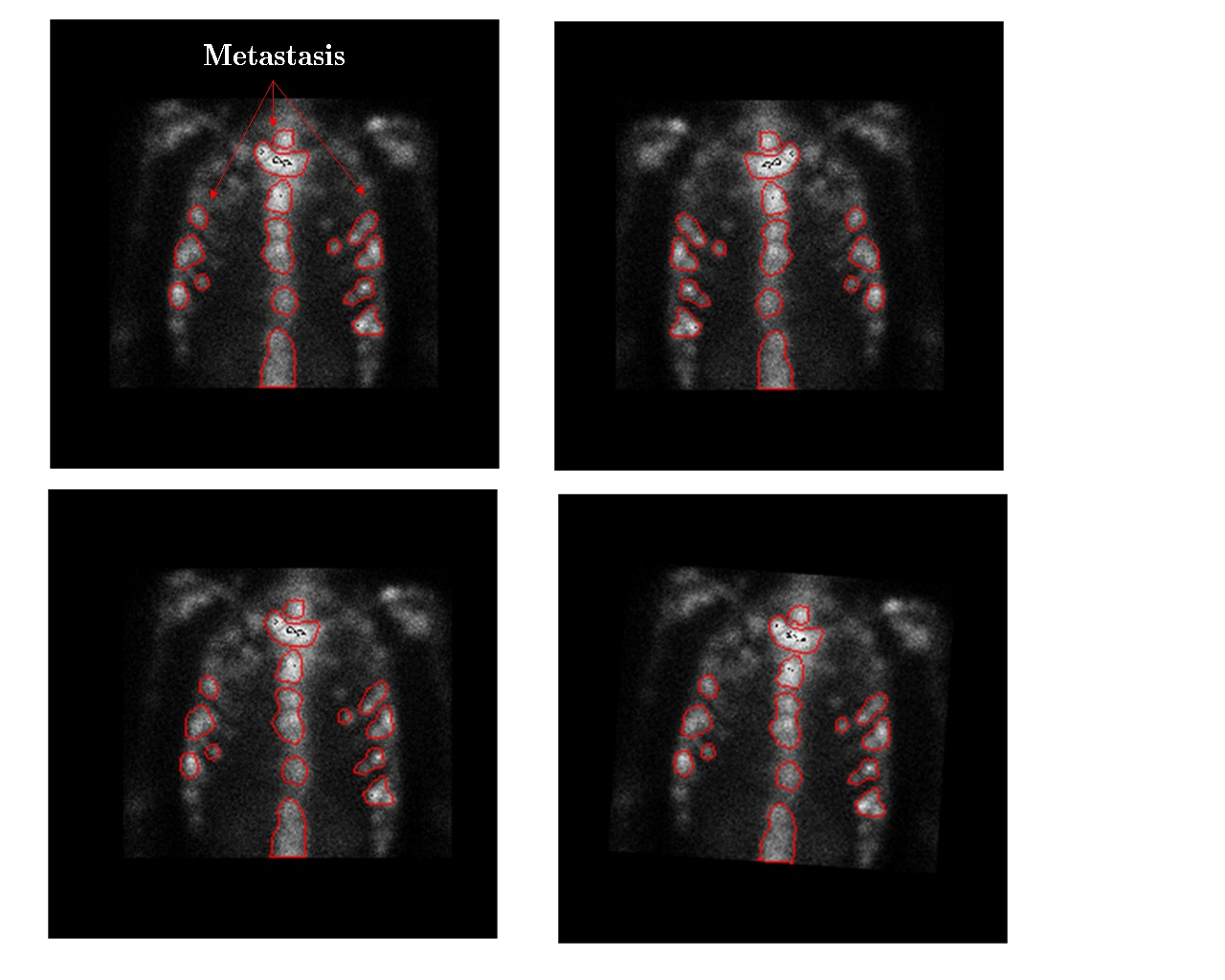

Supplement: S3 Fig — a) The original thorax bone SPECT image; b) The horizontally mirrored image; c) The horizontally translated image by + 6 pixels; and d) The rotated image by +5°. (TIF) [file pone.0243253.s003.tif]

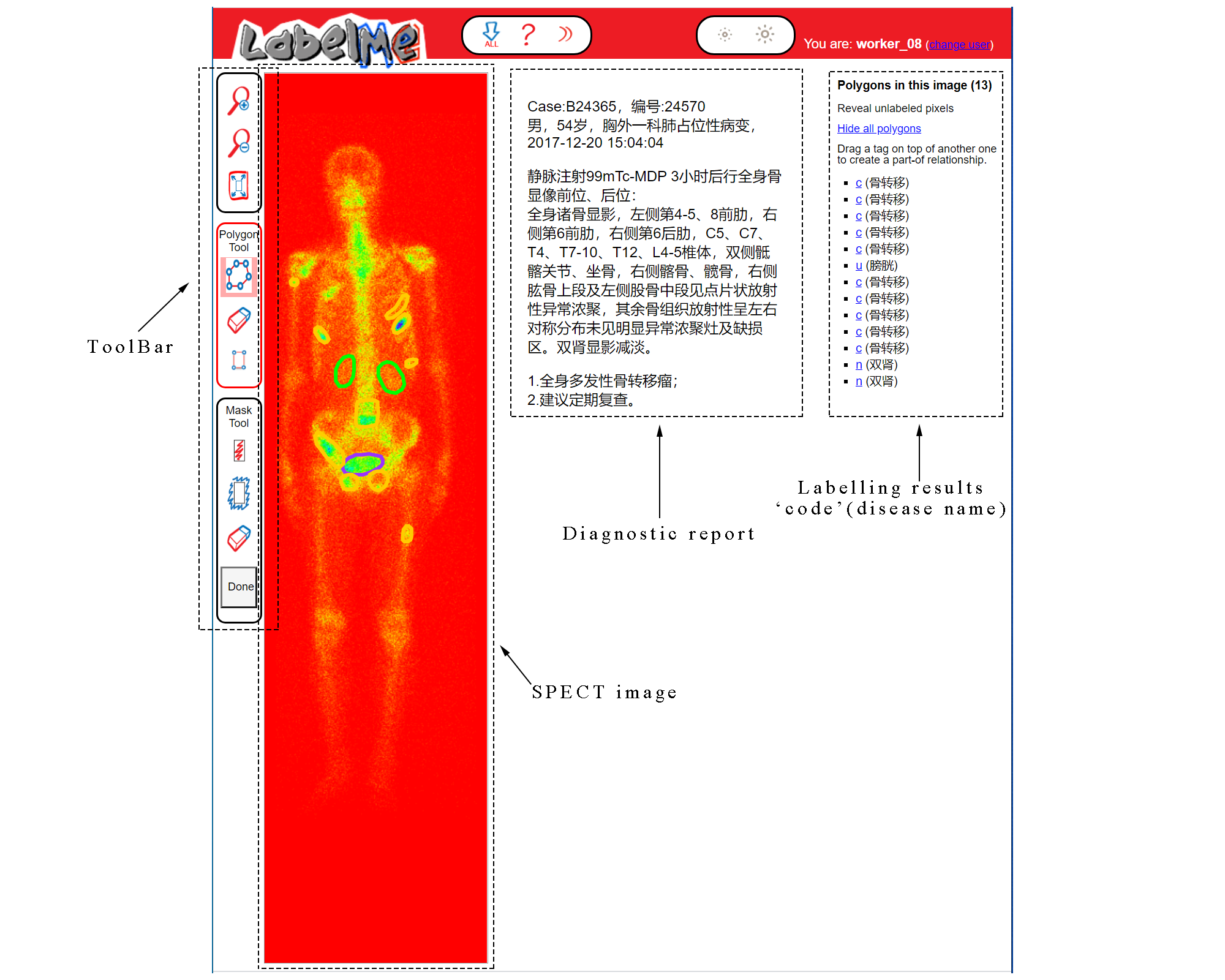

Supplement: S4 Fig — (TIF) [file pone.0243253.s004.tif]

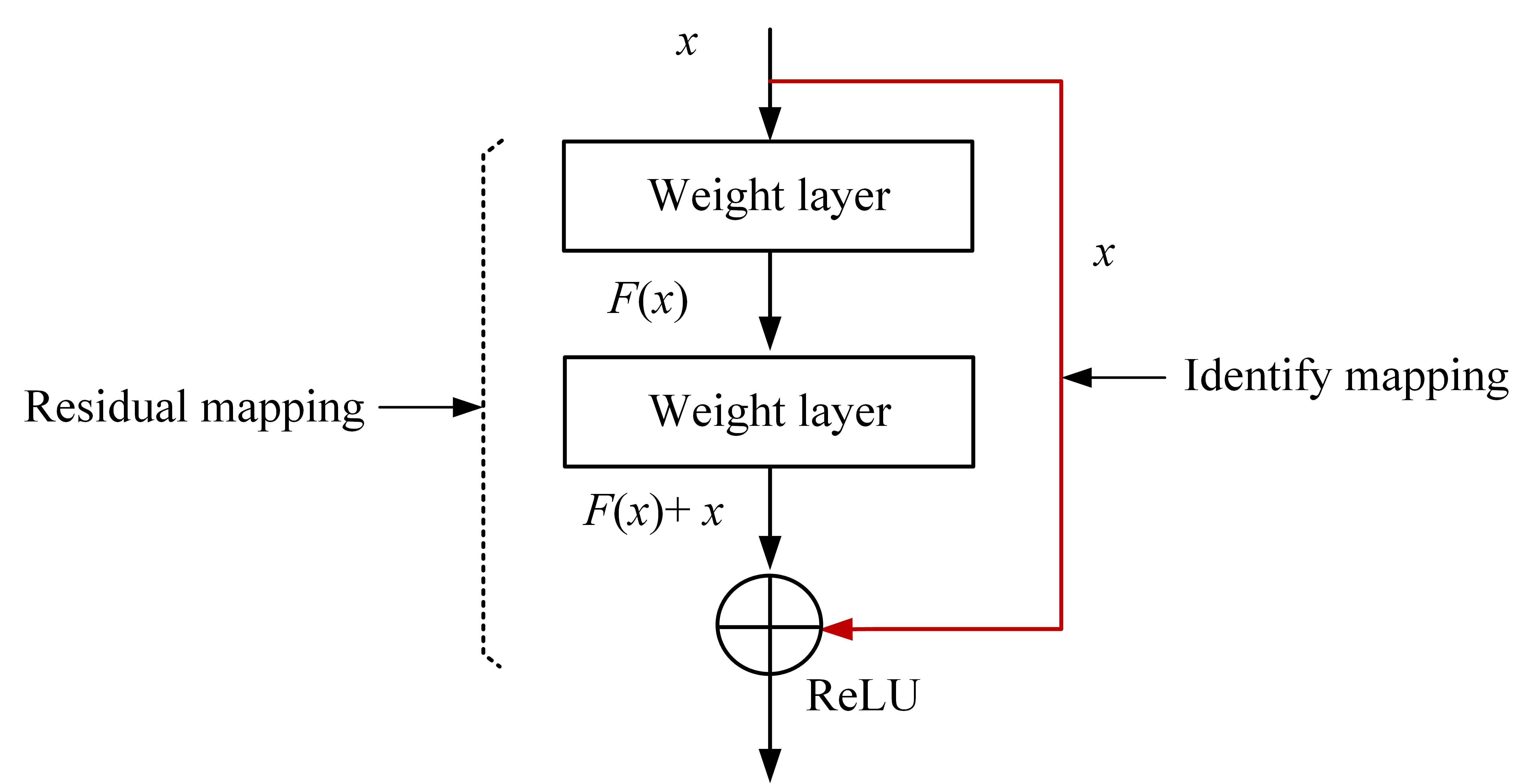

Supplement: S6 Fig — (TIF) [file pone.0243253.s006.tif]

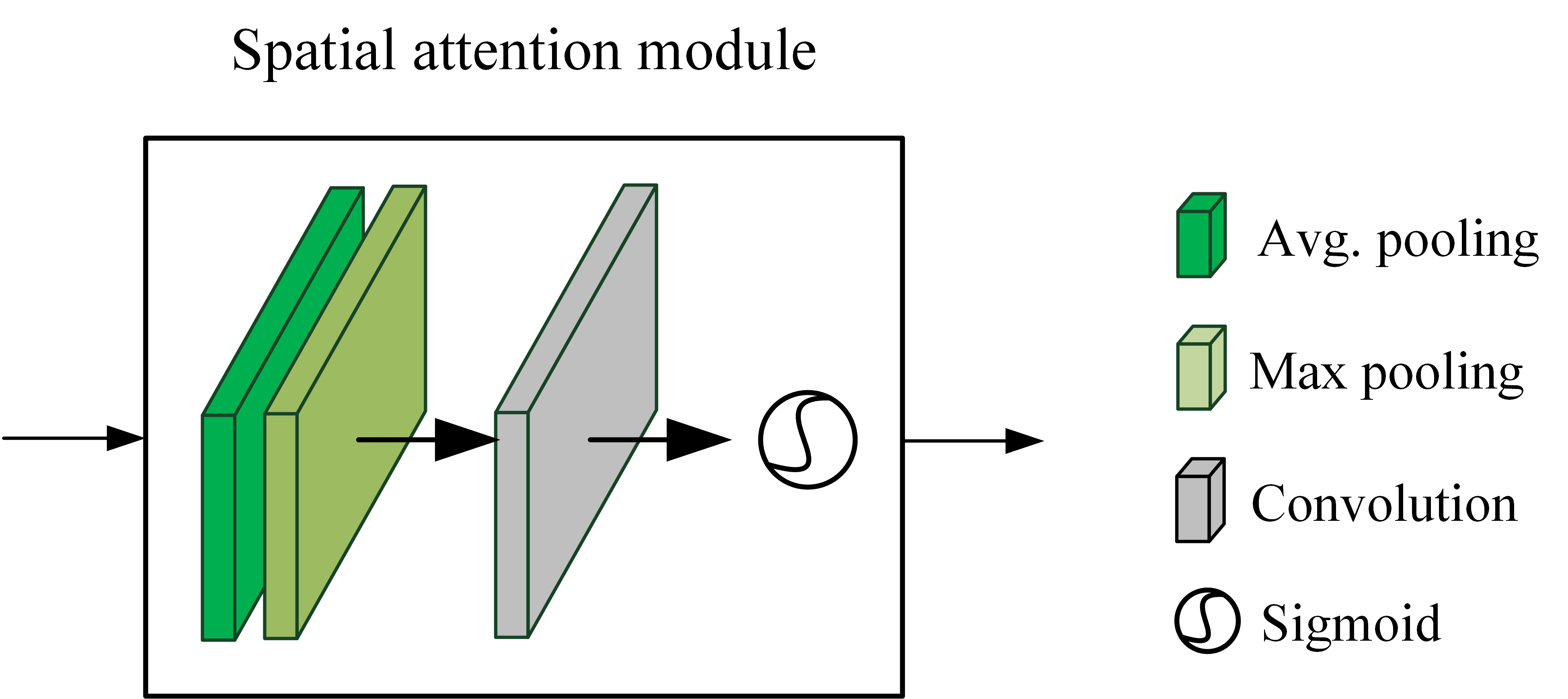

Supplement: S8 Fig — (TIF) [file pone.0243253.s008.tif]

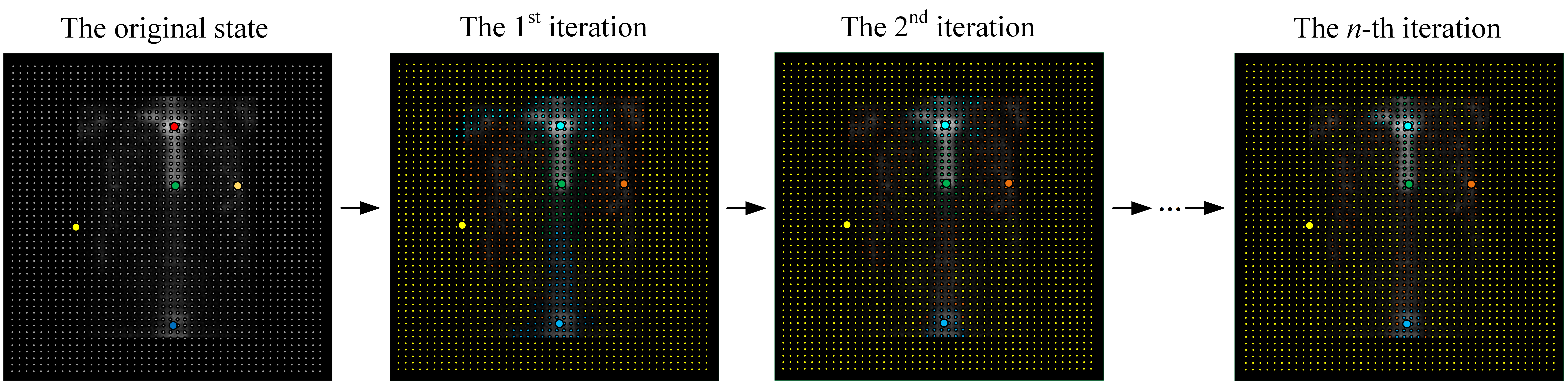

Supplement: S9 Fig — (TIF) [file pone.0243253.s009.tif]

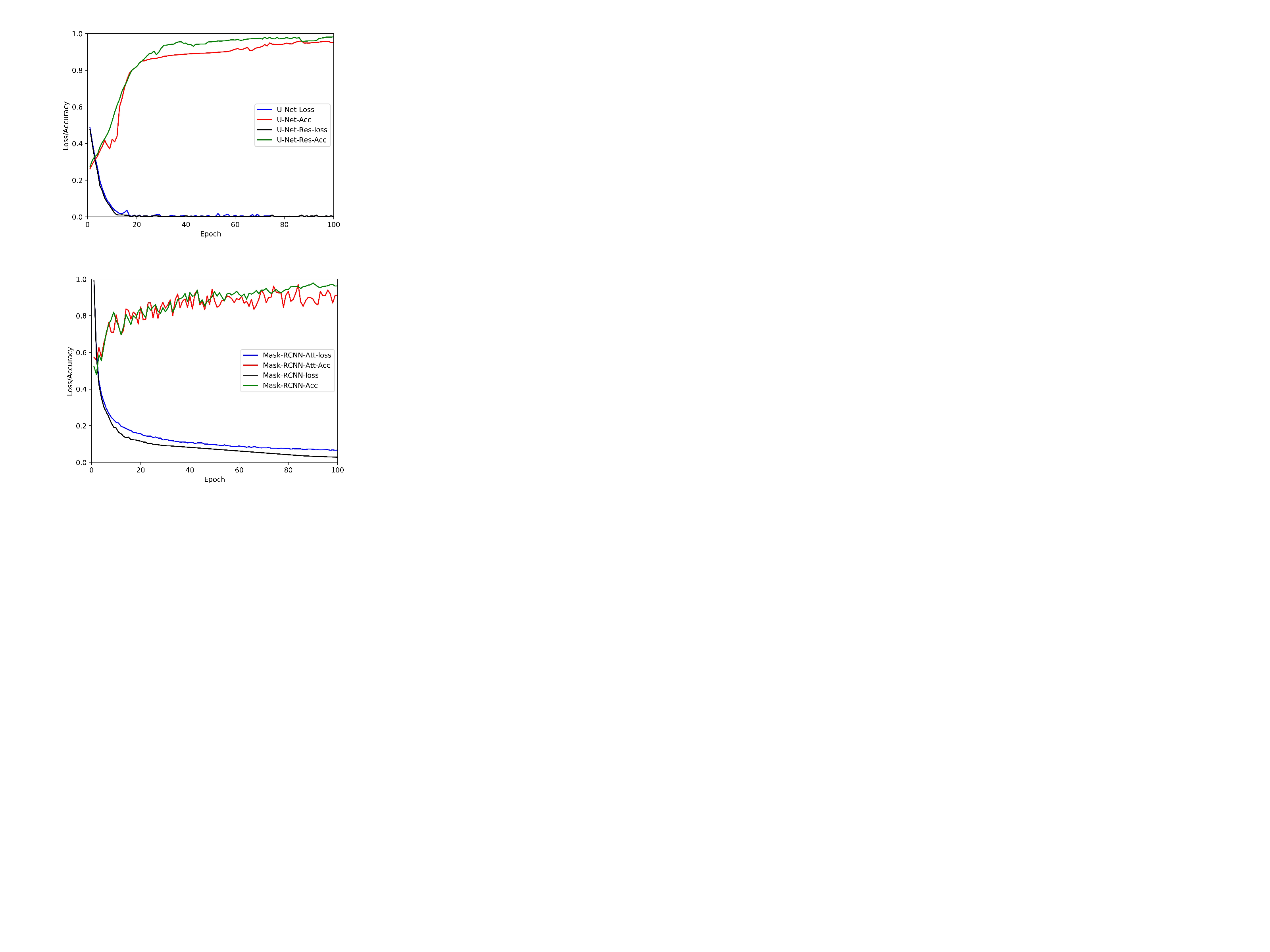

Supplement: S10 Fig — a) U-Net; and b) Mask R-CNN. (TIF) [file pone.0243253.s010.tif]

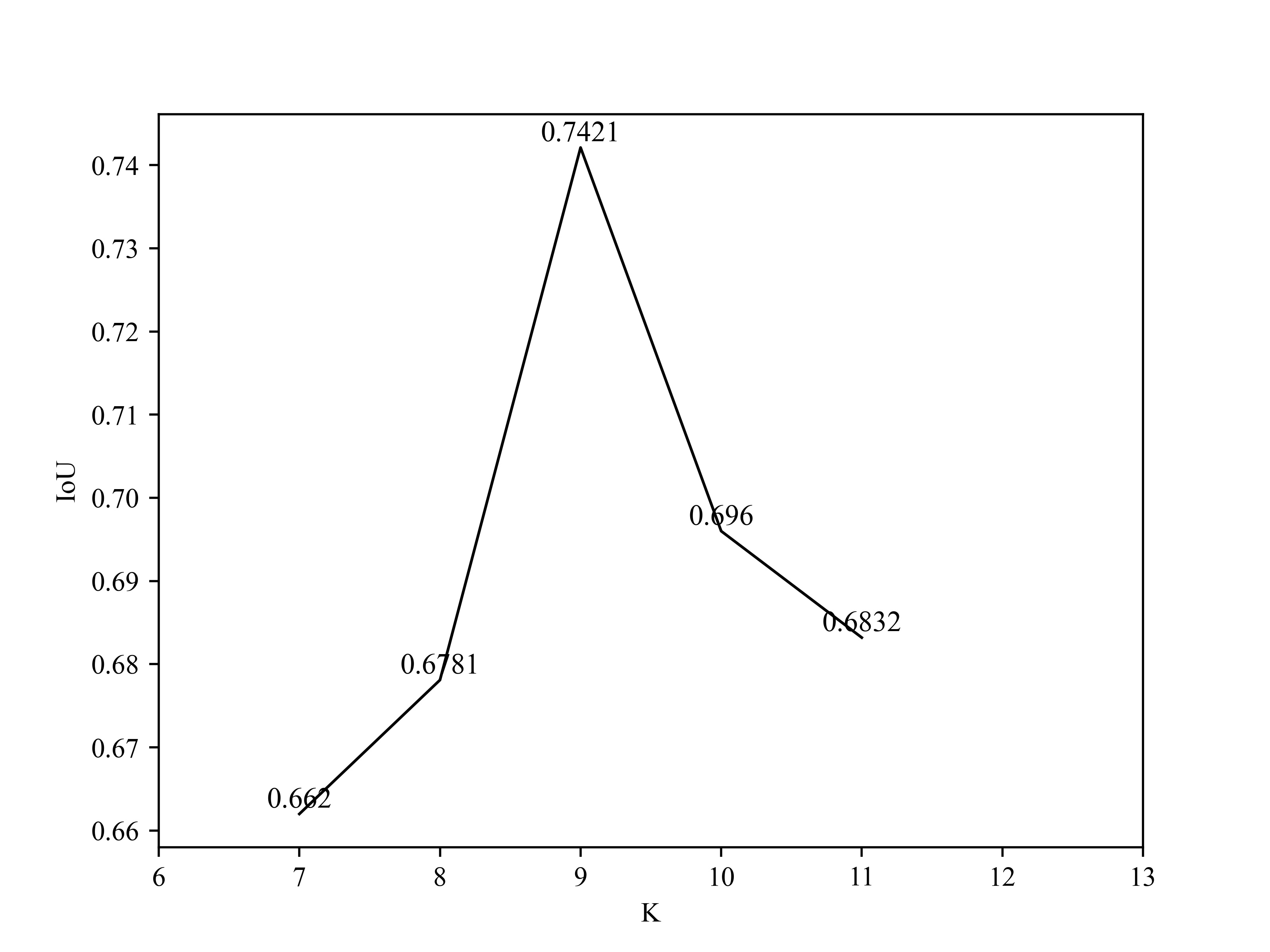

Supplement: S11 Fig — (TIF) [file pone.0243253.s011.tif]

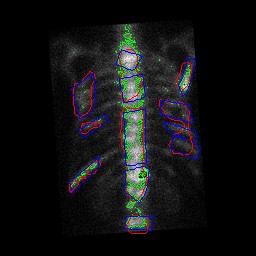

Supplement: S12 Fig — (TIF) [file pone.0243253.s012.tif]

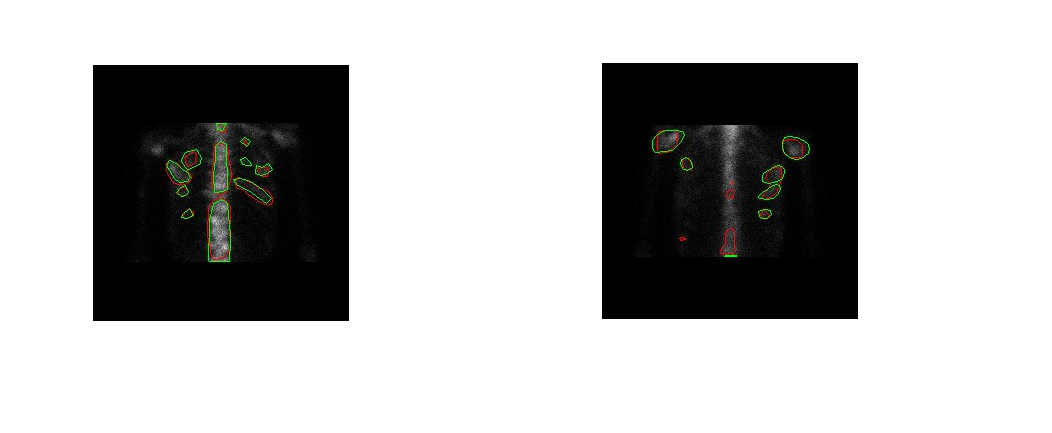

Supplement: S13 Fig — a) The best case; and b) The worst case. (TIF) [file pone.0243253.s013.tif]
