# Supplementary material for: Deep learning based automatic segmentation of metastasis hotspots in thorax bone SPECT images
Source: PLoS One. 2020 Dec 3;15(12):e0243253. doi: 10.1371/journal.pone.0243253 (PMC7714246; doi:10.1371/journal.pone.0243253)
Supplement: S1 Table — (DOCX) [file pone.0243253.s014.docx]

**S1 Table.** An overview of the used data of SPECT images.

| **Dataset** | **Sample** | **Training sample** | **Testing sample** |
| --- | --- | --- | --- |
| **The original** | 112 | – | – |
| **The augmented** | 2 280 | 1 830 | 450 |
