# Supplementary material for: Deep learning based automatic segmentation of metastasis hotspots in thorax bone SPECT images
Source: PLoS One. 2020 Dec 3;15(12):e0243253. doi: 10.1371/journal.pone.0243253 (PMC7714246; doi:10.1371/journal.pone.0243253)
Supplement: S2 Table — (DOCX) [file pone.0243253.s015.docx]

**S2 Table.** Experimental results on evaluation metrics for 2 280 samples of thorax bone SPECT imaging.

| **Segmentation model** | ***PA*** | ***CPA*** | ***Rec*** | **IoU** |
| --- | --- | --- | --- | --- |
| **U-Net** | **0.9920** | 0.7624 | 0.6726 | 0.5941 |
| **U-Net-Res** | 0.9818 | **0.7721** | **0.6788** | **0.6103** |
| **Mask R-CNN** | 0.9724 | 0.7292 | 0.6508 | 0.5544 |
| **Mask R-CNN-Att** | 0.9676 | 0.6958 | 0.6348 | 0.5427 |
